# What impact does medicines shortages have on patients? A qualitative study exploring patients’ experience and views of healthcare professionals

**DOI:** 10.1186/s12913-021-06812-7

**Published:** 2021-08-17

**Authors:** Muhammad Atif, Azka Sehar, Iram Malik, Irem Mushtaq, Nafees Ahmad, Zaheer-Ud-Din Babar

**Affiliations:** 1 Department of Pharmacy Practice, Faculty of Pharmacy, The Islamia University of Bahawalpur, Bahawalpur, Pakistan; 2 Department of Education, The Islamia University of Bahawalpur, Bahawalpur, Pakistan; 3 Department of Pharmacy Practice, Faculty of Pharmacy and Health Sciences, University of Balochistan, Quetta, Pakistan; 4 Department of Pharmacy, University of Huddersfield, Huddersfield, UK

**Keywords:** Medicines shortages, Impact, Access to medicines, Generic substitution, Essential medicines, Drug regulatory Authority of Pakistan

## Abstract

**Background:**

The shortage of medicines represents a complex global phenomenon that triggers patient care and safety issues. The study was undertaken to explore the impact of medicines shortages on patients in Pakistan. The study also identified barriers which hinder the solutions of medicines shortages issue.

**Methods:**

A qualitative study design was adopted and the data was collected in stages between July and September 2019using an in-depth interview approach. The purposive and convenient sampling strategy was used to recruit the study participants. Sample size was limited by using the saturation point criteria. All interviews were audio-recorded, transcribed verbatim, and analyzed using thematic analysis.

**Results:**

A total of 35 stakeholders including 13 physicians, 12 pharmacists and 10 patients participated in the study. The findings of the study were classified into five key themes and seven subthemes. The five themes included, ‘impact of medicine shortages on patients’, ‘patients’ practices in response to medicine shortages’, ‘influence of medicines shortages on medical practice or pharmaceutical business’, ‘barriers to solutions for medicines shortages’, and ‘suggestions to assuage the impact of medicine shortages.’This study showed that the medicine shortages had significant clinical and financial impact on patients. Patients’ opted for a number of risk-prone practices to avoid treatment disruption during shortages. An array of pharmaceutical market, medicines quality and patient related factors refrain physicians to switch from brand name medicine to generics and lead to ineffective management of medicines shortages. Promotion of generic prescription, implementation of punitive policies and proper patient consultation was advised to assuage the impact of medicine shortages on patients.

**Conclusion:**

The adverse clinical, economic and humanistic impact affirmed in this study demand the introduction of risk-management strategies for medicines shortages in hospital and community settings in accordance with the international standards. Promotion of effective patient counselling by the healthcare professionals to deter risk-prone practices associated with medicines shortages is mandatory.

**Supplementary Information:**

The online version contains supplementary material available at 10.1186/s12913-021-06812-7.

## Background

Access to affordable essential medicines is a fundamental human right and Target 3.8 of the Sustainable Development Goals [1]. Nevertheless, according to the World Health Organization (WHO), nearly one-third of the world’s population has limited access to safe, effective and quality pharmacological therapies [2, 3]. Although, the dilemma of medicines shortages is not new, its frequency and severity is intensifying at seemingly inexorable pace [4, 5]. Increasing trends in medicines shortages have been documented in many developed and developing nations, such as the United States (US), the United kingdom (UK), Canada, China, Israel, Australia, Fiji, Finland and Iran etc. [5–14]. Healthcare professionals and healthcare systems around the globe expend millions of hours and hundreds of millions of dollars on the management of medicines shortages [15]. Medicines shortages affect a variety of medicines from various pharmacological groups, ranging from medicines for communicable to non-communicable diseases [5, 9, 16–18]. Contributing factors to medicines shortages emerge mostly from disturbance at any point in the delicate pharmaceutical supply chain.

Considering the complexity and kaleidoscopic nature of the problem, the International Pharmaceutical Federation has advocated country-specific investigations to assess various aspects of medicines shortages in-order to effectively curb them [19]. However, according to the WHO, few low-and-middle- income countries (LMICs) have yet investigated this problem [17]. Moreover, an aspect entirely missing from the literature worldwide is a study of patients’ perspectives on what they actually experience due to the unavailability of medicines, as highlighted by the Food and Drug Administration (FDA) Drug Shortage Task Force Report and recently published literature reviews [3, 15, 20]. Medicines shortages have drastic implications on patients [3, 15, 20]. They are known to contribute towards adverse patient events including treatment delays, clinical complications, and substandard treatment and associated medication errors and adverse drug reactions [21]. Similarly, when medicines are in short supply, their costs may escalate, imposing financial burdens on patients and healthcare institutes [22, 23].

Not an exception, Pakistan has long struggled with inadequate access to essential medicines [4, 24]. Apparently, Pakistan’s healthcare sector is committed to synchronizing legislation with the mandates stipulated in international agreements to guarantee access to quality assured medicines [25]. The timely revision of the National Essential Medicines List for the public health sector in Pakistan is the responsibility of Pharmacy Services, the Drug Regulatory Authority of Pakistan (DRAP) [25]. The rules for the import of raw materials, production, storage, distribution and sale of pharmaceuticals are defined under the Drugs Act, 1976 (XXXI of 1976), and the DRAP guarantees its implementation [26]. Regardless of a reasonably resilient national pharmaceutical framework, the country routinely suffers from shortages of a comprehensive list of medicines for a number of demand and supply related reasons [4, 27–30]. Most recently, the COVID-19 pandemic has more explicitly exposed the vulnerabilities of the pharmaceutical supply chain and legislative protocols [24]. In this regard, medicines shortages have become a major challenge for Pakistani healthcare regulators, as it has considerably undermined the management of patients [24, 31–33]. Furthermore, the use self-medication of substandard alternative medications due to lockdown driven limited access to healthcare professionals has raised the likelihood of medication safety issues [24, 25, 34, 35]. Despite these repercussions, there is currently a limited research data on medicines shortages from Pakistan. Only two published studies – one qualitative [4] and one quantitative [36] – have examined this issue and provided preliminary information about basic characteristics of the predicament, supply related reasons for shortages and have proposed the potential solutions. However, data about consequences of medicines shortages from the patient’s perspective is non-existent. Recognizing the knowledge gap, in-depth exploratory research was undertaken with the aim to explore the direct and indirect impact of medicines shortages on patients. The perspective of patients was sought with the objective to illustrate the consequences they endure. In addition, the viewpoint of physicians and pharmacists was sought with the objective to identify the barriers which hinder the solutions to anomaly.

## Method

### Study setting

This study was conducted in the district of Bahawalpur, Punjab, Pakistan. Bahawalpur is the 12th largest city of Pakistan with an approximate population of 3,333,467 people [37]. To cater to the basic health related needs of the population, Bahawalpur has two tertiary care public sector hospitals; one is the Bahawal Victoria Hospital (BVH) with the capacity of 2200 beds; the second hospital is the Civil Hospital with 410 beds capacity. There are many private sector hospitals in the city, and qualified healthcare practitioners run their private clinics during the evening hours. A total of 500 drug retail outlets – with the majority being operated without the presence of pharmacists – are located in Bahawalpur [38]. In this study, physicians were recruited from the BVH, the Civil Hospital and the private sector hospitals, while the hospital and community pharmacists working in the BVH, the Civil Hospital and the retail pharmacies were included in the study. A large number of patients attend drug retail outlets on daily basis and hence, the patients were recruited from drug retail outlets.

### Study design

A qualitative methodological approach was applied to explore the views of patients, pharmacists and physicians regarding impact of medicines shortages on patients. In-depth interview approach was selected to achieve the study objectives because this approach is suitable to explore untouched issues and effectively translate the interviewees’ viewpoints and experiences [6]. The Consolidated Criteria for Reporting Qualitative Research (COREQ) 32-item checklist [39] was followed to present the findings of this study (Supplementary File 1).

### Selection of study participants

Purposive sampling accompanied by convenience sampling was used to engage participants [40]. In the first stage, all healthcare professionals (HCPs) were purposively approached during working hours through telephone call or personal visit and invited to participate after explaining the brief intent of the study. Additional information concerning the study was provided to them on demand. In the second step, those who consented to participate in the study were face-to-face interviewed at their offices, residences, restaurants or any other place convenient to them. To ensure the reliability and quality of the data gathered, the physicians and pharmacists having a work experience of ≥3 years and ≥ 1 year, respectively, were invited to participate in the study. Participation was also sought from the patients attending the drug retail outlets to purchase their medicines with or without prescription, having any disease, and had experienced medicines shortages in the past. Those who consented to participate and had no communication difficulties were interviewed on the spot or at the time and place convenient to them, for example, during evening hours at their residence or restaurant.

### Interview schema development

The interview schema (Supplementary File 2) for each stakeholder was designed to respond to the study rationale after comprehensive literature review [4, 9, 41] and consultations with the experts. The pilot interviews with two representatives each from the three stakeholders were conducted before the final interviews to ensure validity, uniformity and understandability of the study protocols [6]. The interview questions began with general questions, and then narrowed down to the study’s key objectives.

### Data collection

The data were collected in stages between July and September 2019. In the first stage, physicians and pharmacists were interviewed from July to August 2019. In the second stage, patients were interviewed during September 2019. All interviews were conducted in Urdu (the national language of Pakistan), and were recorded on an audio tape. The senior authors frequently debriefed the interviewing author (AS), to ensure the standardized administration of the interview guide, and thereby enriching the quality and trustworthiness of the data. The sample size was determined by applying the saturation point criteria [6]. A total of three additional interviews (one from each group of participants) were conducted to remove doubts in the data saturation. At the end of each interview, field notes were also taken instantly and impressions were recorded.

### Data analysis

The data from HCPs and patients were separately subjected to thematic analysis [42]. The audio recorded interviews were listened carefully and transcribed word for word by AS. Verbatim English translation of all the transcribed interviews was undertaken. The audio recordings of the interviews were listened iteratively and the written transcripts were also read repeatedly to gain a rich and deep understanding of the data. The meaningful words, phrases and sentences related to study’s objectives were extracted manually from each interview after considerable discussion with research supervisor, and a list of initial codes was generated (IM and AS). Following initial coding, focused coding explored the relationship between different initial codes. The final codes were subsequently grouped into meaningful categories (IM). Themes and subthemes were generated by bringing several categories together (MA, IM, IMU, NA and ZB). Cross-checking was undertaken to ensure data credibility and enhance the trustworthiness of the data. In case of any conflict or disagreement, the verdict given by the supervisor (MA & IMU) was deemed final.

## Results

### Characteristics of participants

A total of 43 participants (i.e., 15 physicians, 15 pharmacists and 13 patients) were invited to participate in the study. Of these, seven refused due to their busy schedule, while 35 agreed to participate in the study. In all, we conducted interviews with 13 physicians and 12 pharmacists and 10 patients. Among 13 physicians, nine were male and four were female. Pharmacists were distributed equally in each gender category (i.e., six males and six females). Of the total 10 patients, six were male and four were female, and half had secondary level of education. The average duration of interviews with physicians, pharmacists and patients was 24 min (SD = 3.2), 28 min (SD = 3.7) and 22 min (SD = 3.4), respectively. Physicians and pharmacists had an average experience of 7.9 years and 3.8 years, respectively. The respondents’ characteristics are given in Table 1.

**Table 1:** Characteristics of study participants

Characteristics of healthcare professionals			Characteristics of patients	
	Physicians (*n* = 13)	Pharmacists (*n* = 12)		*n* = 10
**Gender**			**Gender**	
Male	9	6	Male	6
Female	4	6	Female	4
**Experience (years)**			**Educational level**	
Average	7.9	3.8	Secondary	5
Range	3–15	1–15	Intermediate	1
**Interview duration (minutes)**			Graduate	2
Average (SD)	24 (3.2)	28 (3.7)	Postgraduate	1
Range	20–32	22–43	Masters	1
			**Interview duration (minutes)**	
			Average (SD)	22 (3.4)
			Range	18–30

**Fig. 1:**
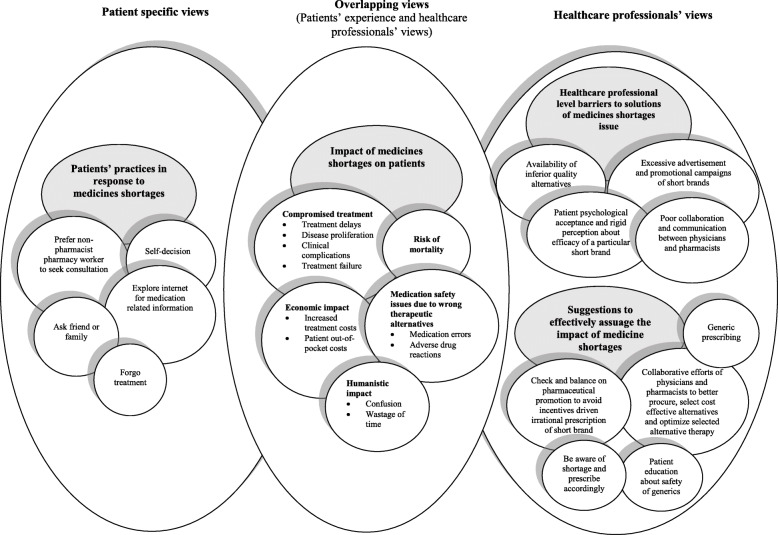
The summary of findings

The analysis of the data extracted from interviews yielded five key themes and seven subthemes. The themes covered ‘impact of medicine shortages on patients’, ‘patients’ practices in response to medicine shortages’, ‘influence of medicines shortages on medical practice and pharmaceutical business’, ‘barriers to solutions of medicines shortages issue’, and ‘suggestions to assuage the impact of medicine shortages’. The summary of the findings is provided in Fig. 1.

### Overlapping theme

#### Theme 1: impact of medicines shortages on patients

Almost all the study participants had health related concerns about the patients. According to them, medicines shortages adversely impact patients. From a clinical point of view, most HCPs (23 out of 25 HCPs) highlighted compromised treatment (20 out of 25 HCPs), medication safety issues (18 out of 25 HCPs) and risk of mortality (14 out of 25 HCPs) as the consequences of medicine shortages. According to them, medicine unavailability contributed to treatment delays, disease proliferation and complications, and treatment failure. Further, they elaborated that shortages can place patient at increased risk of medication safety, such as wrong therapeutic alternatives, medication errors and adverse drug reactions. Likewise, patients reported compromised patient care and treatment delays (7 out of 10 respondents), adverse events (8 out of 10 respondents) and high risk of mortality (9 out of 10 respondents) when questioned about whether they had experienced any consequences because of medicines shortages. In addition to the clinical impact, both HCPs (21 out of 25 respondents) and patients (9 out of 10 respondents) implied that shortage cause financial burden on patients, and humanistic impact in the form of confusion and wastage of time.

**Fig. 2:**
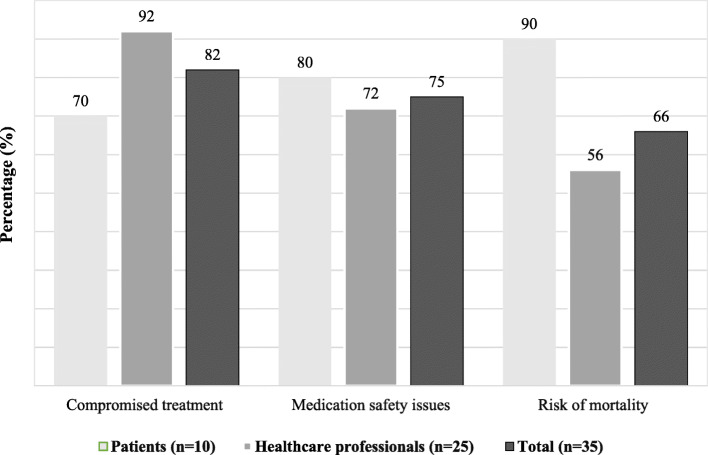
Responses of each of participants concerning impact of medicines shortages

The overlapping theme (Theme 1), respective subthemes and categories, together with illustrative quotes are presented in Table 2. Figure 2 provides graphical presentation of impact of medicines shortages by each set of participants.

**Table 2:** Impact of medicines shortages on patients (overlapping theme)

Themes	Subthemes	Categories	Supporting quotations
Impact of medicines shortages on patients	Clinical impact	Compromised treatment • Delayed treatment • Disease proliferation and complications • Treatment failure	*“Obviously, when patient fails to take medicine according to prescribed regimen, his disease proliferates and it could be difficult to control again. This is very evident in case of infectious diseases and cancer treatment. When good efficacy antibiotic brand is not available, recovering the patient becomes difficult.”* (Physician 4) *“Sometimes efficacy of alternative generic as compared to international brand is less and that is unable to properly treat a disease.”* (Pharmacist 1) *“It has huge impact on the patient. Patient suffers more than anyone else. I have experienced this in my skin allergy and the brand was not available due to shortage. Even cream and soap were not available. I was very worried because I wanted to recover quickly. Although, alternative brands were available, but I just wanted that specific brand. Because there was an ingredient in that brand, which was more effective for my skin.”* (Patient 5)
		Medication safety issues due to • Wrong therapeutic alternative • Medication error • Adverse drug reactions • Other risk associated with substandard and counterfeit medicines	*“When any brand is short, we have to change it or sometimes, new brand has different strength or dosage form. As a result, the treatment regimen changes, but our patients are used to follow previous regimen and mostly have limited literacy level. In such case, risk of medication error increases and very often patient return and complain that they are having side effects.”* (Physician 8) *“There are also few antihuman elements and when prescribed brand is not available, they take benefit from it and start selling substandard and counterfeit medicines. This happens especially with branded medicines because few brands have high demand. These counterfeit medicines are harmful and cause adverse drug reactions…”* (Pharmacist 5) *“Alternative brands are mostly available but then there can be strength issues and it impacts patient a lot. I have blood pressure issue and I use Byscard 2.5 mg. Few days back, I don’t know it was short or dispenser was careless but he gave me 5 mg and he didn’t guide me to take half tablet. Due to which my blood pressure got very low, which was not good impact. I could have died.”* (Patient 4)
		Risk of mortality	*“In hospitals, there are many patients who cannot afford a single penny over medicines, so ultimately, when they cannot get medicines from hospitals, their condition gets worsen and worsen or some may lead to death as well.”* (Physician 4) *“If any generic gets short then obviously all those patients who are taking that generic, for example asthma patients have to take inhalers, TB patients have to be on ATT so their whole treatment will be disturbed, even this could be fatal…”* (Pharmacist 6) *“Sometimes, it turns into a matter of life and death. My father is heart patient and you know anti-angina medicine is very much important for such patients. But it was critically short in the market. I am pharmaceutical distributor but it was difficult even for me to find it. You can understand the consequences if such important medicines are short...”* (Patient 7)
	Humanistic impact	• Confusion • Wastage of time	*“Comparable efficacy brands are available in markets, but as most of the people are illiterate, they are not aware of it. If the physician prescribes him a brand which is not available in the market, he will be confused and faced with a problem in search of that brand. Patients waste time and ask for same medicine brand again and again, because they want that specific medicine brand.”* (Physician 11) *“Patient experiences a lot of confusion when prescribed brand is not available in the market. If a pharmacist advises patient on some alternative brand, he usually hesitates to take his advice and proceed to search for the particular brand.”* (Pharmacist 5) *“If there is a shortage of branded medicine then we have to visit other pharmacies. It takes a lot of time. In case alternative is available, we have the satisfaction issue that whether the alternative will be good or not, we will recover or not. We feel the need to visit the physician again to confirm.”* (Patient 8)
	Financial burden	• Increased treatment costs • Patient out-of-pocket costs	*“If we give alternative and that alternate is costly then it effects on the pocket of the patient.”* (Pharmacist 13) *“In case of shortage, patients buy medicines from drug retail outlets. But usually, patients who visit government hospitals are not rich and they only come in search of free medication. When any medicine is not available in the hospital, we ask them to buy it from outside and this increases burden on patients.”* (Physician 9) *“It takes a considerable amount of time and money. Sometimes, we have to purchase high price alternative brand or the same brand at high price from black marketers.”* (Patient 8)

### Patients’ specific theme

#### Theme 2: patients’ practices in response to medicine shortages

When asked whom they consult in order to avoid disruption of their treatment during medicines shortages, five patients reported seeking physician consultation. The majority of the patients (8 out of 10 patients) preferred non-pharmacist pharmacy workers for consultation, and only (4 out of 10 patients) reported having consulted a pharmacist amid shortages. Patients asserted that they prefer non-pharmacist pharmacy workers because of their good knowledge and way of medication related counseling. Other practices opted by a plethora of patients included self-decision about alternative brands (8 out of 10 patients), exploring the internet (6 out of 10 patients), asking for family or friend advice (5 out of 10 patients) and forgoing treatment (3 out of 10 patients). The patient specific theme (Theme 2), respective subthemes and categories, together with illustrative quotes are presented in Table 3.

**Table 3:** Patients’ practices in response to medicine shortages (patients’ specific theme)

Themes	Subthemes	Categories	Supporting quotations
Patients’ practices in response to medicine shortages	Preference for type of healthcare professional to seek advice when medicine is not available	• Few preferred physicians • Very few preferred pharmacists • Majority preferred non-pharmacist pharmacy worker	*“I ask physician because he is a person authorized to do so. So, I take medicine that he prescribes. Because best practice is, medicine should be prescribed by the person who has diagnosed the disease.”* (Patient 5) *“If there is a pharmacist in the pharmacy, then I usually ask him for the alternative brand. At big pharmacies, pharmacist is very cooperative and they guide me about my medicine and help me understand how to take it”* (Patient 6) *“I seek advice from dispenser. Dispensers know everything about medicines, their brands and prices and they are better than pharmacists and physicians. They even know where that medicine will be available in the market...”* (Patient 2)
	Other approaches	Self-decision	*“Or if there is general fever or pain medicine shortage then I take any available brand because I know all such medicine work the same”* (Patient 9)
		Ask family or friends	*“… If any family member has same illness or is in the medical field then I ask them…”* (Patient 7)
		Forgo treatment	*“When dispenser tells me about the shortage of the brand I asked for, then I know it will be not available at other pharmacies in the vicinity as well. I don’t travel to any other store and try to treat myself with home remedies.”* (Patient 4)
		Explore using internet	*“I Google to match the ingredients between the prescribed brand and dispensed brand. If there is no difference, I consume the medicine fearlessly.”* (Patient 3)

### Healthcare professionals’ specific themes

#### Theme 3: influence of shortages on medical practice and pharmaceutical business

When physicians were asked if they had encountered challenges in the event of medicines shortages, nine out of 13 physicians reported personal dissatisfaction and loss of their credibility among patients. From an economical point of view, the majority of HCPs (20 out of 25 HCPs) indicated that shortages of medicines often lead to financial losses for providers and pharmaceutical business. They were of the view that financial losses due to consistent short medicines sometimes compel them to switch to alternative brand to avoid same loss in future. However, this may impact patient management due to differences in efficacy and lack of patient’s psychological acceptance or dissatisfaction.

#### Theme 4: barriers to solutions of medicines shortages issue

According to the healthcare professionals, an array of barriers contributed to ineffective solution of medicines shortages problem, thereby negatively influencing the desired patient outcomes. Major reasons that refrained physicians from switching to alternative generics during shortages included excessive advertisement and promotional campaigns of short brands (25 out of 25 HCPs) and availability of inferior quality alternatives (24 out of 25 HCPs). Further, stakeholders indicated that it was challenging for them to minimize the impact of shortages by switching to alternate brand because of patient psychological acceptance and rigid perception about efficacy of a particular short brand. With regard to hospitals, many healthcare professionals (15 out of 25 HCPs) reported difficulties in correctly selecting and procuring important medicines and the consequent inexorable shortage of high demand medicines due to poor collaboration and communication between physicians and pharmacists.

#### Theme 5: suggestions to assuage the impact of medicine shortages

Stakeholders reported a number of potential ways to assuage the impact of medicine shortages. Among these, the key suggestion for addressing medicines shortages was enactment of generic prescription in the country or prescription of medicines by international nonproprietary name (INN) (24 out of 25 HCPs), and check and balance on pharmaceutical promotion to avoid incentives driven irrational prescription of short brand (22 out of 25 HCPs). Upon further exploration, the participants indicated the physician as having the responsibility to offset the issue through educating patients about the efficacy of alternative brands (18 out of 25 HCPs), and staying up-to-date on the unavailability of any brands in the market (13 out of 25 HCPs). With regard to management of shortages in hospitals, collaborative efforts of physicians and pharmacists to better procure, select cost-effective alternative brands and optimize selected alternative therapy were stressed by more than half of the respondents (14 out of 25 respondents).

All healthcare professionals’ specific themes (i.e., Theme 3, Theme 4 and Theme 5), respective subthemes and categories, together with illustrative quotes are presented in Table 4.

**Table 4:** Healthcare professionals’ specific themes

Themes	Subthemes	Categories	Supporting quotations
Negative influence of shortage on practice and pharmaceutical business	Impact on physicians	• Patient dissatisfaction • Personal dissatisfaction	*“Its effects physician’s credibility. If physician prescribes a short brand to patient, patient thinks that physician is taking incentives for this brand that’s why he is prescribing this despite shortage.”* (Physician 12) *“Physician faces problem, as he loses confidence. When he usually prescribes some trustworthy brands but if he has to switch to other brands or local brands due to shortage, then he remains uncertain that whether it has same efficacy and gives same results or not, my patient will be satisfied or not…”* (Physician 5)
	Impact on business	• Credibility loss • Financial loss	*“Impact on the pharmacy business depends on brands whose shortages occur because some pharmaceutical companies provide more margins to pharmacies for specific brands. So if those well-known brands get into shortage, then there will be profit loss.”* (Pharmacist 5) *“Financial losses are obvious and then there are credibility losses. One of the major losses is that your customers are dependent on your drug. So your relations with them are disturbed. So both financial and credibility losses happen.”*(Pharmacist 4)
Healthcare professional level barriers to solutions of medicines shortages issue		• Excessive advertisement and promotional campaigns of short brands	*“Incentives play a key role here, if pharmaceutical companies provide incentives to create demand of their product. They do good marketing and continually approach physicians. As a result, physician will prescribe more of their short brand and patient suffers.”* (Physician 7) *“According to medical representatives, customer is not patient but a physician. They directly convince the physician that if they prescribe short brand then they will get reward or incentives in return. In such cases, patient continues to experience due to unavailability of medicines. If physicians easily switch to alternative, they can manage the shortages very easily.”* (Pharmacist 7)
		• Inferior generics	*“Secondly, in Pakistan the quality also matters, if there are multinationals then they are of good quality while the local are not of high quality sometimes.so there is an issue. Different brands have different quality; good brands show good effect like we have omeprazole of Esomepra, it is very much better as compared to any other company’s brand.”* (Physician 6) *“One issue is efficacy of alternatives. Local companies don’t conduct comparative bioequivalence studies and there is no data about its efficacy. Second many pharmaceutical companies use substandard excipients to manage the price of product. As a result, quality of generic products is poor as compared to branded medicines.”* (Pharmacist 1)
		• Poor collaboration and communication between physicians and pharmacists	*“Major issue is poor collaboration, which leads to poor demand prediction in hospitals. Demands are not based on actual needs of patients. Medicines having very low demand are purchased in large quantities and medicines having high demand are purchased in small quantities. We cannot blame administration for this. It is the duty of pharmacists and physicians to decide which medicine is needed in large quantities, but as you know physicians and pharmacists do not collaborate much in our hospitals.”* (Pharmacist 5)
		• Patient psychological acceptance and rigid perception	*“It’s not related to physicians but is based on patient satisfaction of its own. It is also based on patient psychology that if they are satisfied with a specific brand then they think they will be harmed by switching to generic. So, they resist changing brand, demand the same.”* (Physician 7) *“Patient trust physician so he sets his mind on brands prescribed by physician. He thinks physician is always right. For instance, there was a case when Calpol was prescribed by the physician and I dispensed Panadol to the patient due to shortage, but he refused to take that. Its patients’ psychological issue that they think physician has prescribed this brand, so we have to take this, otherwise we would not get treated.”* (Pharmacist 10)
Suggestions to effectively assuage the impact of medicine shortages		• Generic prescribing/ Prescribing by international nonproprietary name (INN)	*“If government changes its policy and promotes generic prescription instead of brand, and compels companies to market medicines with the generic name only then branded shortage issue can be managed or entirely vanished.”* (Physician 7) *“There should be generic prescription, like in European countries. Even in Pakistan, there is only generic prescription in some high-level hospitals, such as Shaukat Khanum hospital and Agha khan hospital. But, there is brand prescription in government or some other hospitals due to incentives. Because incentives are only received on brands and not on generics. If they shift to generic, then all brand authority will shift to pharmacist. Pharmacist will be responsible for brand selection. When prescription comes with generic, pharmacist will provide patients with the best brand available.”* (Pharmacist 9)
		• Check and balance on pharmaceutical promotion to avoid incentives driven irrational prescription of short brand	*“Physicians should not stick to one brand because of any agreement. Companies should be punished for luring physicians through incentives.”* (Physician 1) *“Again I would say that the government should keep an eye on promotional practices of pharmaceuticals. Also they should bound physicians not to make deals with the companies. If physicians are strictly punished for being biased towards a brand, this issue will vanish by itself…Physicians need to work ethically and prioritize patients rather than personal gains.”* (Pharmacist 12)
		• Be aware of shortage and prescribe accordingly	*“If a medicine is not available, physician should be aware of shortage. If he knows then he should shift his patients to alternate brand as soon as possible.”* (Pharmacist 2) *“Sometimes, physicians don’t have information about brand shortage. If medical representatives have not informed the physician, then he will continue to prescribe that brand.”* (Physician 8)
		• Patient education about safety of generics	*“If any patient is taking one brand of antihypertensive or anti-diabetic, then physician should educate him that other brands are also available. Whether patient is educated or not, educates him as much as you can; that this medicine is available under different brand names, but salt is same in each brand. This brand suits you but in case if shortage of that brand occurs then you can switch to another brand....”* (Physician 1) *“Patients should have knowledge of brands if one brand is short in market then there is always an alternative of that brand available. Based on the mind of our patients, it is the duty of physicians to spread awareness among patients about alternative brands”* (Pharmacist 11)
		• Collaborative/mutual efforts of physicians and pharmacists to better procure, select cost effective alternative and optimize selected alternative therapy	*“If we don’t know which alternative brand is better or at least equivalent in terms of efficacy then pharmacist should guide us. Also he should tell us all the possible side effects if they suggest any new salt because many times we resist switching just to avoid negative impact on patient.”* (Physician 1) *“The collaboration between physician and pharmacists should be promoted, because as I said before procurement of medicines in hospitals is heavily affected due to this issue and leads to shortage.”* (Pharmacist 5)

## Discussion

The consequences of medicines shortages are challenging, especially for the patients and healthcare system of LMICs, including Pakistan [14, 36, 41, 43]. However, according to the FDA, there is a scarcity of research data on the consequences of medicines shortages [20]. Therefore, a qualitative approach was adopted predominantly to explore the impact of medicines shortages on patients in Pakistan from the viewpoint of patients and healthcare professionals. The study findings highlighted the clinical, economic and humanistic impact of medicine shortages on patients. An important aspect uncovered was the practices of patients to avoid interruption of their treatment or the wrong selection of alternative treatment during medicines shortages. In addition, healthcare professionals’ specific themes highlighted the impact of shortage on medical practice or pharmaceutical business, barriers to solutions for medicines shortages, and corresponding suggestions.

The participants in this study reported many clinical implications of medicines shortages. According to them, the impact on patients could be altered dosage regimen, disease proliferation and complications, medication errors, adverse drug events and high risk of mortality due to inappropriate alternatives, and substandard and counterfeit medicines. Correspondingly, all patients interviewed in this study confirmed that they had experienced these consequences owing to shortages. These were fully supported by published scoping reviews sought to assess the impact of medicines shortages on patient outcomes [3, 15]. Beyond increasing the supply of medicines to avoid treatment disruptions, healthcare regulators should consider introducing a prospective risk assessment system – which involves assessment of utilization patterns of the medicines affected, and the appropriate protocols for substituting them –in hospital and community settings to prevent patient harm. The same has been advocated bythe American Society of Health System Pharmacists, the Therapeutics Goods Administration, and the European Medicine Agency on managing shortages [44].

In addition to clinical consequences, the study participants reported medicines shortages being a problem for patients in terms of increased treatment costs and out-of-pocket expenses. These findings highlighted more pronounced clinical impact of medicines shortages on patients due to increased likelihood of abandoned treatment, as up to 25% of Pakistani people live below the poverty line and cannot bear out-of-pocket expenses [45, 46]. Similarly, a number of studies from Australia, Europe, South Africa and Canada had reported economic impact of shortages on patients [8, 43, 47, 48]. Other negative outcomes reported by our study participants included patients being confused while selecting alternate brand, and waste of time in ensuring the correct selection of brand [25]. These findings also accord with the findings of the US studies [49, 50]. Physicians and pharmacists also reported having economic and credibility losses when they were unable to meet the expectations or demands of their patient/customers. These implications of medicines shortages were also highlighted by studies from Fiji and Europe [14, 23, 43, 51].

Another important theme that arose from the data was patients’ practices in response to medicines shortages. A plethora of patients reported that they preferred consultation with non-pharmacist pharmacy workers followed by consultation with physicians and pharmacists, to prevent harm and interruption of their treatment. The reason for their inclination towards non-pharmacist pharmacy workers may be due to ease of access to dispensers, high consultation fees of physicians, and lack of awareness about pharmacists driven by their limited availability in hospital and community settings [25, 52]. However, considering the poor medication related knowledge and training of non-pharmacist pharmacy workers, this indicated a significant safety concerns for the consumers [52, 53]. Moreover, the patients in this study reported self-decision regarding alternative medicine, practice of exploring the internet for medicine related information, seeking advice from family or friend, and not taking medicines in response to medicines shortages. Worryingly, these practices coupled with poor health literacy and high prevalence of inappropriate use of medicines may potentially exacerbate the impact of medicines shortages on patients [25, 26, 54].

In this study, several barriers contributing to the ineffective solution of medicines shortages and relevant suggestions were also reported by the healthcare professionals. According to them, major reasons that refrain physicians from switching to alternative generics during shortages were excessive advertisement and promotional campaigns of those brands which were short in supply. In several instances these brands were of inferior quality. These barriers are attributable to lax regulatory infrastructure, the unavailability of national guidelines for the prescription of generics, and the questionable quality of generics owing to the lack of bioavailability or bio-equivalence studies [4, 55, 56].

In this regard, the study participants recommended generic prescribing, regulatory check and balance on promotional agreements between pharmaceutical companies and physicians, and selection of better alternative in collaboration with pharmacists. These suggestions were also strongly advocated in previous studies from Pakistan, Malaysia and Taiwan [4, 55–58]. Furthermore, stakeholders indicated that it was challenging for them to minimize the impact of shortages by switching to alternate brands because of patient psychological acceptance and firm belief in a particular brand. In addition to the practices of the practitioner, the preferences of patients play a pivotal role in medicine selection and the probability of switching brands [59]. To this end, the study participants indicated that physicians can offset the impact of shortages by staying aware of the unavailability of a specific brand and by prescribing available generics along with proper patient education about its efficacy. A good body of evidence has emphasized the need for patient education to promote generic drugs [60–64]**,** thereby remedying shortages**.** With regard to hospitals, healthcare professionals suggested improved collaboration and communication between physicians and pharmacists. This was reportedly because of the need to better procure, and select the alternative therapy. The importance of this suggestion can be gauged by the fact that pharmacists around the world are utilizing their problem-solving skills and collaborating with prescribers and nurses in hospitals during COVID-19 pandemic. They preserve medicines prone to shortage by identifying possible alternatives, optimizing selected therapy and reducing wastage of important medicines [65–67]. However, in Pakistan, the longstanding inadequate participation of hospital pharmacists in medication related decisions is attributable to the limited number of hospital pharmacists, as well as their derisory acceptance by physicians [53, 68–70].

This study successfully filled out the gap particularly concerning patient experiences regarding medicines shortages, and views of physicians and pharmacists on the impact of medicines shortages in Pakistan. However, there are a few limitations in this study. First, the participants in the study were all from one city. Second, some important stakeholders, for example, nurses, manufacturers, health regulators and distributors were not included in this study. Third, the findings of this preliminary study, though have provided solid foundation for future research, might have understated or overstated the impact of medicines shortages due to biasness. However, to generate more reliable evidence, it is strongly advised that future studies should involve all stakeholders from different cities and perform broad quantitative surveys to visibly estimate the impact of medicines shortages on patients.

## Conclusion

This study revealed that access to medicines in Pakistan continues to be problematic with adverse clinical, economic and humanistic effects on patients. Patients opt for a number of risk-prone practices to avoid treatment disruption during shortages. An array of pharmaceutical market, medicines quality and patient related factors refrain physicians from switching brand to available generic medicines, which in turns leads to ineffective resolution of medicines shortages. Promotion of generic prescription, active involvement of pharmacists in healthcare delivery system, implementation of effective policies and proper patient consultation are advised in this context.

### Impact of findings on policy and practice

In addition to the implications of this study for the literature, insights about various aspects can be gleaned from the findings and may help concerned stakeholders to justify legislative change and interventions.

Taken as a whole, the adverse consequences of medicines shortages urgently require the introduction of risk-assessment and risk-management strategies in hospital and community settings in accordance with the international standards. Moreover, a harmonized impact scoring system is mandatory to streamline the efforts targeted at medicines shortages issue. To deter risk-prone patient practices, patients should be deftly counseled by the prescribers and made aware of the generics, management approaches they should follow, and to whom they should communicate during shortages.

In order to minimize the influence of medicines shortages resulting from excessive promotional campaigns on solution to medicines shortages, strict enforcement of punitive polices and logical implementation of generic prescribing policy could be considered on priority basis.

The study findings strongly advocate future large scale quantitative studies to measure the extent and overall harm of medicines shortages in order to inform decision makers and address the problems

## Supplementary Information

**Additional file 1.** COREQ checklist.

**Additional file 2.** Interview schema of physicians, pharmacists and patients.

## Data Availability

The data will be made available upon receiving reasonable request. The requests may be directed to the corresponding author.

## Abbreviations

BVH: Bahawal Victoria Hospital
COREQ: The Consolidated Criteria for Reporting Qualitative Research
DRAP: Drug Regulatory Authority of Pakistan
FDA: Food and Drug Administration
LMICs: Low-and-middle- income countries
US: United States
UK: United Kingdom
WHO: World Health Organization
