# Supplementary material for: What impact does medicines shortages have on patients? A qualitative study exploring patients’ experience and views of healthcare professionals
Source: BMC Health Serv Res. 2021 Aug 17;21:827. doi: 10.1186/s12913-021-06812-7 (PMC8369330; doi:10.1186/s12913-021-06812-7)
Supplement: Supplementary file 1 — Additional file 1. COREQ checklist. [file 12913_2021_6812_MOESM1_ESM.docx]

**Supplementary File 1: COREQ checklist**

| **No. Item** | **Guide questions/description** |  | **Location in Manuscript (Section and Page #)** |
| --- | --- | --- | --- |
| **Domain 1: Research team and reﬂexivity** |  |  |  |
| *Personal Characteristics* |  |  |  |
| 1. Inter viewer/facilitator | Which author/s conducted the interview or focus group? | AS | Author contributions |
| 2. Credentials | What were the researcher’s credentials? E.g. PhD, MD | M.Phil. | Title page |
| 3. Occupation | What was their occupation at the time of the study? | Full time student. | - |
| 4. Gender | Was the researcher male or female? | Female | - |
| 5. Experience and training | What experience or training did the researcher have? | She has attended training sessions in qualitative studies. | - |
| *Relationship with participants* |  |  |  |
| 6. Relationship established | Was a relationship established prior to study commencement? | Yes. | - |
| 7. Participant knowledge of the interviewer | What did the participants know about the researcher? e.g. personal goals, reasons for doing the research | Participants were encouraged to read the purpose of the study and the confidentiality statement before starting the interview. The researcher explained that she is collecting data for educational purpose. | Declarations – 34 |
| 8. Interviewer characteristics | What characteristics were reported about the inter viewer/facilitator? e.g. Bias, assumptions, reasons and interests in the research topic | It was explained that this research is being conducted for academic purpose. No interviewer related biasness was identified. | - |

| **Domain 2: study design** |  |  |  |
| --- | --- | --- | --- |
| *Theoretical framework* |  |  |  |
| 9. Methodological orientation and Theory | What methodological orientation was stated to underpin the study? e.g. grounded theory, discourse analysis, ethnography, phenomenology, content analysis | Thematic analysis. | Method – 8 |
| *Participant selection* |  |  |  |
| 10. Sampling | How were participants selected? e.g. purposive, convenience, consecutive, snowball | Convenient and purposive sampling. | Method – 7 |
| 11. Method of approach | How were participants approached? e.g. face-to-face, telephone, mail, email | Face to face interviewed. | Method – 7 |
| 12. Sample size | How many participants were in the study? | 35. | Result – 10 |
| 13. Non-participation | How many people refused to participate or dropped out? Reasons? | Seven eligible participants refused because of their busy work schedule. | Result – 10 |
| *Setting* |  |  |  |
| 14. Setting of data collection | Where was the data collected? e.g. home, clinic, workplace | Participants were interviewed at a place comfortable to them which included their home and cafeteria at their work place. | Method – 7 |
| 15. Presence of non-participants | Was anyone else present besides the participants and researchers? | No. | - |
| 16. Description of sample | What are the important characteristics of the sample? e.g. demographic data, date | Yes | Results – 10, 11 |
| *Data collection* |  |  |  |
| 17. Interview guide | Were questions, prompts, guides provided by the authors? Was it pilot tested? | Yes. | Method − 8 |
| 18. Repeat interviews | Were repeat interviews carried out? If yes, how many? | There were no formal repeat interviews. However, after listening the recordings again and again, few participants were contacted to clarify or further explain their responses. | - |
| 19. Audio/visual recording | Did the research use audio or visual recording to collect the data? | Interviews were audio recorded. | Method – 8 |
| 20. Field notes | Were ﬁeld notes made during and/or after the interview or focus group? | Yes. | Method – 8 |
| 21. Duration | What was the duration of the inter views or focus group? | The average duration of interviews with doctors, pharmacists and patients was 24 minutes (SD=3.25), 28 minutes (SD=3.73) and 22 minutes (SD=3.42), respectively | Results – 10 |
| 22. Data saturation | Was data saturation discussed? | Yes. | Method – 8 |
| 23. Transcripts returned | Were transcripts returned to participants for comment and/or correction? | No. Participants were offered to read the transcripts but none of them were willing. | - |
| **Domain 3: analysis and ﬁndings** |  |  |  |
| *Data analysis* |  |  |  |
| 24. Number of data coders | How many data coders coded the data? | All the authors (MA, AS, IM, IMU, ZB) except NA participated in data coding | Authors’ contributions – 35 |
| 25. Description of the coding tree | Did authors provide a description of the coding tree? | Yes. | Method – 8, 9 |
| 26. Derivation of themes | Were themes identiﬁed in advance or derived from the data? | Derived from the data. | Method – 8, 9 |
| 27. Software | What software, if applicable, was used to manage the data? | None. | - |
| 28. Participant checking | Did participants provide feedback on the ﬁndings? | No one was interested to provide feedback | - |
| *Reporting* |  |  |  |
| 29. Quotations presented | Were participant quotations presented to illustrate the themes/ﬁndings? Was each quotation identiﬁed? e.g. participant number | Yes. | Table 2 – 4 |
| 30. Data and ﬁndings consistent | Was there consistency between the data presented and the ﬁndings? | Yes. | - |
| 31. Clarity of major themes | Were major themes clearly presented in the ﬁndings? | Yes. | Discussion – 29 to 32 |
| 32. Clarity of minor themes | Is there a description of diverse cases or discussion of minor themes? | Yes. | Discussion – 29 to 32 |
