# Supplementary material for: What impact does medicines shortages have on patients? A qualitative study exploring patients’ experience and views of healthcare professionals
Source: BMC Health Serv Res. 2021 Aug 17;21:827. doi: 10.1186/s12913-021-06812-7 (PMC8369330; doi:10.1186/s12913-021-06812-7)
Supplement: Supplementary file 2 — Additional file 2. Interview schema of physicians, pharmacists and patients. [file 12913_2021_6812_MOESM2_ESM.docx]

**Supplementary File 2**

**Interview schema of doctors**

| **Discussion topic** | **Probes** |
| --- | --- |
| **General Information** | 1. Respondent ID……………… 2. Gender ……………. 3. Age …………… 4. Years of medicine related working experience…………. |
| 1. **During the last year, have you encountered or has your patient reported unavailability of prescribed brand in the market?** | - If yes, please list several names of those brands……... |
| 1. **How frequently do you encounter the shortage of branded medicines?** |  |
| 1. **What is the overall impact of branded medicines shortage on patients?** | - What types of impact are there? - What is the severity of impact? |
| 1. **Is there any impact of brand medicines shortage on your professional practice?** |  |
| 1. **If some specific brands are short in the market, then what physicians do to ensure the continuity of treatment?** |  |
| 1. **Do you find it difficult to manage patient when required brand is not available?** | If yes, what sort of difficulties do you face? |
| 1. **In your opinion, how the impact of branded medicines shortages can be minimized?** |  |

**Interview schema of pharmacists**

| **Discussion topic** | **Probes** |
| --- | --- |
| **General Information** | 1. Respondent ID……………… 2. Gender ……………. 3. Age …………… 4. Years of medicine related working experience…………. |
| 1. **During the last year, have you encountered unavailability of prescribed brand?** | - If yes, please list several names of those brands……... |
| 1. **How frequently do you encounter the shortage of branded medicines?** |  |
| 1. **What is the overall impact of branded medicines shortage on patients?** | - What types of impact are there? - What is the severity of impact? |
| 1. **Is there any impact of brand medicines shortage on your professional practice or pharmaceutical business?** |  |
| 1. **If some specific brands are short in the market, then what physicians do to ensure the continuity of treatment?** |  |
| 1. **Do you find it difficult to manage patient when required brand is not available on pharmacy?** | - If yes, what sort of difficulties do you face? |
| 1. **In your opinion, how the impact of branded medicines shortages, especially on patients, can be minimized?** |  |

**Interview schema of patients**

| **Discussion topic** | **Probes** |
| --- | --- |
| **General Information** | 1. Respondent ID……………… 2. Gender ……………. 3. Age………………. 4. Education level………………. 5. Place of residency………… |
| 1. **What do you know about branded medicine and its shortage?** |  |
| 1. **Have you ever experienced brand shortage?** | - If yes - Then which brand? |
| 1. **What is the impact of branded medicines shortage on patient?** | - What impact you or your family members have experienced? - What is the severity of those impact? |
| 1. **If brand prescribed by doctor is not available, then what you do to ensure the continuity of your treatment?** | - What approach do you adopt? - Whom do you prefer to consult when in doubt? - Do you face difficulties while managing? If yes, explain |
| 1. **If your physician prescribed a branded medicine but pharmacist/pharmacy worker offered you another brand, what you do?** | - Do you satisfactorily follow the advice of pharmacist/pharmacy worker? If not, why? |
| 1. **How patients should overcome branded medicines shortages issue in your opinion to avoid any bad consequences?** |  |
